# A Rapid Procedure for the Simultaneous Determination of Eugenol, Linalool and Fatty Acid Composition in Basil Leaves

**DOI:** 10.3390/foods11213315

**Published:** 2022-10-22

**Authors:** Lucia Lenti, Daniela Rigano, Sheridan L. Woo, Ancuta Nartea, Deborah Pacetti, Filippo Maggi, Dennis Fiorini

**Affiliations:** 1 Chemistry Division, School of Science and Technology, University of Camerino, Via Madonna delle Carceri 9/B, 62032 Camerino, Italy; 2 Department of Pharmacy, School of Medicine and Surgery, University of Naples Federico II, Via Domenico Montesano 49, 80131 Naples, Italy; 3 Institute for Sustainable Plant Protection, National Research Council, 80055 Portici, Italy; 4 Department of Agricultural, Food and Environmental Science, Polytechnic University of Marche, 60131 Ancona, Italy; 5 School of Pharmacy, University of Camerino, Via Madonna delle Carceri 9/b, 62032 Camerino, Italy

**Keywords:** basil, eugenol, linalool, fatty acids, simultaneous analysis, quality control

## Abstract

Eugenol and linalool are often the most abundant volatile compounds found in basil (*Ocimum basilicum* L., Lamiaceae) leaves, and they are interesting for the aroma they provide and for their numerous beneficial bioactivities. Their determination is thus needed for several purposes. In the present study, to avoid the previous isolation of essential oil, the direct solvent extraction is proposed coupled with a transmethylation to convert acyl lipids into fatty acids methyl esters (FAMEs), thus assessing the possible simultaneous analysis of eugenol and linalool with FAMEs by gas chromatography coupled to flame ionization detector (GC-FID). The method has been validated and applied to ten basil leaves samples in which eugenol and linalool were found in mean concentrations of 2.80 ± 0.15 and 1.01 ± 0.04 g kg^−1^ (dry weight), respectively. FAMEs composition was dominated by linolenic acid (52.1–56.1%) followed by palmitic acid (19.3–22.4%) and linoleic acid (9.6–11.3%). The ratio of *n*6-polyunsaturated fatty acids (PUFAs)/*n*3-PUFAs was in the range of 0.17–0.20 in the investigated samples. The proposed method exploits a rapid procedure requiring 40 min, making use of a small amount of solvent and allowing the simultaneous determination of molecules contributing to assess the quality of this worldwide appreciated herb.

## 1. Introduction

Basil is an annual plant native to subtropical regions from central Africa to Southeast Asia, cultivated nowadays worldwide. Known as sweet basil also referred to as “king of the herbs”, it is in the foreground among the aromatic herbs, having mostly culinary uses, but also applications in aromatherapy, perfumery, cosmetics, traditional medicine, food supplement production. All parts of the plant are aromatic. The ovate, slightly toothed and glabrous leaves are arranged oppositely along the square stems and the small flowers, usually white or pale purple, in whorls at the nodes of the inflorescence. The fruit consists of four nutlets. Leaves, in fresh or dry form, are the part of the plant more commonly used for culinary purposes due to their aroma given by the volatile components. Essential oil (EO) content and composition vary greatly among the different varieties according to the different morphological structures characterizing the different varieties. The volatile components are produced and stored in the glandular trichomes of basil leaves and flowers. The typical aroma of basil is given mostly by monoterpenes and phenylpropenes [1]. 

Linalool and eucalyptol are among the major monoterpenes imparting sweet/floral and fresh/eucalyptus-like aroma, respectively. Eugenol and methyl chavicol are the major phenylpropenes imparting clove and anise-like aroma, respectively [2,3]. 

Beyond the aromatic properties provided by EO making the leaves exploitable for several culinary purposes, basil EO has also been recognized to have antimicrobial, antiulcer, antioxidant, insecticidal, antifungal, anti-inflammatory, immunomodulatory, and insect repelling properties [4]. The reason for these EO activities has often been attributed to linalool [5] that results to be among the major components of basil EO [6] and to phenolic compounds, mostly eugenol and methyl chavicol [7]. Eugenol, due to its antimicrobial properties, has huge commercial worth, expected to reach US$754.1 million revenue by 2026 [8].

Since these components are key volatile components of basil, being the most abundant ones in some chemotypes, they represent important quality markers, and their content is of pivotal importance to estimate the price of basil. In addition, they are endowed with important protective biological activities. Linalool has been reported to have anticancer, antimicrobial, neuroprotective, anxiolytic, antidepressant, hepatoprotective, renal and lung protective activity [9], while eugenol has shown antioxidant, antimicrobial, anesthetic, anti-inflammatory, neuroprotective, anti-diabetic, and anti-cancer properties [10]. Thus, they have application in different sectors.

A classic procedure can be performed extracting these molecules by using an organic solvent due to their lipophilicity. However, this would lead to also extracting the lipid fraction that, even if at low concentration, is found in the sample. Thus, a direct gas chromatography (GC) analysis without any purification treatment should be avoided due to the limited volatility of lipid components. A possibility to overcome this problem could be the previous isolation of EO. However, EO distillation is laborious and requires 2–4 h depending on the chemical composition. Furthermore, sometimes distillation could not allow a complete recovery of the volatiles from the matrix, thus providing only an indication of the content of volatile substances. Fatty acid (FA) composition of the extract is important when considering the potential of an herb from the nutritional, nutraceutical, cosmetic or several other applicative points of view. To the best of our knowledge, only Tarchoune et al. [11] and, more recently, Vlaicu et al. [12] reported on the FA composition of basil leaves. In the first study, EO and FA compositions were evaluated. The authors determined FA profile by GC after lipid extraction and derivatisation of FAs as fatty acid methyl esters (FAMEs), while EO composition was determined after EO isolation by hydro-distillation and subsequent GC analysis. In the second study, FA composition, together with cholesterol and the mineral, polyphenols and nutritional profile of basil, thyme and sage were evaluated to assess their possible exploitation for the development of new functional foods of animal origin.

The study here presented is aimed to propose a method to analyse volatile components found in basil leaves, in particular eugenol and linalool, avoiding the isolation of EO and simultaneously determining FA profile. These analyses performed with classical procedures require a rather long time, i.e., for the distillation to isolate essential oil and then for GC analysis of the volatile components; for the lipid extraction, the derivatisation of acyl lipids as FAMEs, and then their GC analysis, for a total that could be estimated at 4–5 h depending on different parameters such as the distillation time (during which volatiles composition can also change [13]) and the time of GC analysis. To combine the two analyses in a simultaneous and much more rapid procedure, the present study exploits transmethylation of acyl lipids found in the extract to obtain FAMEs that, as opposed to triglycerides and to other acyl lipids derivatives, can be analysed by GC directly and simultaneously with the volatile components present in the sample. Such a method would allow the simultaneous determination of components having an important role in the determination of the basil leaves’ beneficial properties.

## 2. Materials and Methods

### 2.1. Reagents and Standards

Linalool and eugenol pure standards were purchased by Sigma Aldrich. A certified reference standard mixture composed by 37 FAMEs was purchased from Supelco (Bellefonte, Pennsylvania). Sodium chloride was purchased from Carlo Erba (Milan, Italy), sodium sulphate from Fluka (North Carolina, USA), and potassium hydroxide from Merck, (Darmstadt, Germany). Hexane was purchased from Sigma Aldrich (Milan, Italy) while methanol from Fisher (Milano, Italy). Deionized water (resistivity above 18 MΩ cm) was obtained from Milli-Q SP Reagent Water System (Millipore, Bedfort, MA, USA).

### 2.2. Sample Preparation: Extraction and Transmethylation

Sweet basil (*Ocimum basilicum* L. cv Genovese) was used in this experiment, carried out in a protected greenhouse of the BiPaF Section of the Department of Agriculture, University of Naples Federico II, Portici (NA, Italy). Commercial seedlings were transplanted to the field in June 2019. In July, the plants were cut above the first node. Five plants were harvested per replicate. Fresh leaves were quickly frozen in liquid nitrogen to obtain a powder. Basil powder (300 mg) was weighted in a 4 mL screw cap vial, then 50 mg of NaCl and 1 mL of ultrapure water were added to suspend the powder. A volume of 2 mL of hexane was added to the sample and the mixture was vigorously stirred with the help of a vortex device for 2 min. The two phases were allowed to stratify and separate with the use of a centrifuge for 5 min (5000 rpm) and the upper organic phase was collected in a 4 mL screw cap vial. A second extraction was performed with the same procedure and with 1 mL of hexane. The final collected organic phase was dried with anhydrous Na_2_SO_4_. For the subsequent transmethylation, a volume of 0.5 mL of the basil powder extract was transferred in a 2 mL screw cap vial. Subsequently, 0.05 mL of a methanolic KOH solution (2 N) were added and the mixture was vigorously stirred for 2 min. Then, 1 mL of an aqueous solution of acetic acid (0.15 M) was added and the sample stirred with the vortex device for 1 min. The two layers were separated with the help of a centrifuge for 5 min (5000 rpm). The upper organic phase was finally collected and ready to be directly analysed by GC coupled with flame ionization detector (FID). The injection volume was 0.5 µL.

### 2.3. Lipid Fraction Extraction and Transmethylation

The extraction of lipid fraction was performed according to the Folch procedure [14]. For the transmethylation step, 5 mg of lipid extract were weighted in a 2 mL screwed cap vial and dissolved in 1 mL of hexane. Then, 0.1 mL of a methanolic KOH solution (2 N) were added and the mixture stirred for 2 min. Subsequently, 1.5 mL of an aqueous solution of acetic acid (0.15 M) were added and the sample stirred again for 1 min. The two layers were let stratifying with a centrifuge (5 min, 500 rpm). The upper organic phase was collected and a volume of 0.5 µL used for the injection at GC-FID.

### 2.4. Gas Chromatography Analysis

The GC analysis of linalool, eugenol and FAMEs was performed using a GC Agilent Technologies 6850 (Santa Clara, CA, USA) equipped with a split/splitless injector and coupled with an FID. The injection was performed in split mode (split ratio 30:1) with the injector temperature maintained at 260 °C. The carrier gas was hydrogen produced by a generator (Whatman, Model 75–32, from Whatman International Ltd., Milano, Italy) and the initial hydrogen flow in the column was 2.50 mL min^−1^. The capillary chromatographic column was a polar (50%-cyanopropylphenyl)-methylpolysiloxane column (DB225-MS, length 30 m, 0.25 mm i. d., 0.25 µm film thickness, purchased from Agilent Technologies, Santa Clara, CA, USA). The oven temperature was set at 40 °C and maintained for 3 min, then raised at 25 °C min^−1^ until reaching 240 °C and held for 1 min for a final run time of about 12 min. The FID temperature was set at 250 °C and hydrogen and air flows were 40 mL min^−1^ and 400 mL min^−1^, respectively. The identification of analytes was performed by comparing retention times of standard solutions and confirmed by the analysis carried out with a 6890 N GC coupled with a 5973 N single quadrupole mass spectrometer detector (Agilent Technologies, Santa Clara, CA, USA), comparing both retention times and mass spectra of the analytes with those of analytical standards and comparing experimental mass spectra also with those reported in NIST library (2017).

The injector temperature was set at 260° and the injection (1 µL) was performed in split mode (split ratio 40:1). The initial oven temperature was maintained at 40 °C for 3 min, then raised to 15 °C min^−1^ until reaching 240 °C and held for 2.67 min for a final run time of 19 min. The initial carrier gas (helium) flow rate in the column was 1.2 mL min^−1^. The mass analysis was performed in SCAN mode in the range 29–400 Da. The transfer line was maintained at 260 °C, ion source at 230 °C and quadrupole at 150 °C.

### 2.5. Quantification and Method Validation

Standard stock solutions for linalool and eugenol were prepared at a concentration of 10 µL mL^−1^ by dissolving 100 µL of pure standard and adding hexane in a volumetric flask until obtaining 10 mL of solution. The quantification of linalool and eugenol was performed by using linear calibration curves obtained by the injection of 5 standard solutions of the analytes in the range of 50–400 µL L^−1^. For both linalool and eugenol, calibration curves were obtained with linear regression coefficients of 0.999 (Table 1). Limit of Detection (LOD) and Limit of Quantification (LOQ) were defined by considering the peak areas corresponding to 10 and 3 times the S/N ratios, respectively. The obtained values were low enough to permit the detection and quantification of the analytes of interest. In fact, LOD is 0.05 g kg^−1^ for linalool and 0.07 g kg^−1^ for eugenol in the dried powder, while LOQ values are 0.18 and 0.22 g kg^−1^, respectively, much lower than the quantities found in the samples under investigation. For the recovery test, the standard mixture of linalool and eugenol was added to the sample at a known concentration. The dried basil sample was spiked with 10 µL of a standard mixture containing linalool at 25 mL L^−1^ and eugenol at 50 mL L^−1^, then it was subjected to the procedure reported in Section 2.2 and analysed by GC-FID. The recoveries were calculated by the following equation:

(1) $$Recovery\;\left(\%\right)=\frac{\left(Css-Cus\right)}{Cps}\cdot 100$$

where:

*Css* = concentration of the spiked samples, *Cus* = concentration of the unspiked sample, *Cps* = concentration spiked to the sample (Table 1).

### 2.6. Statistical Analysis

Data were submitted to one-way analysis of variance (ANOVA) and to Turkey’s test for pairwise comparison to underline statistically significant differences (*p* < 0.05) between the different extraction methods. PAST software was used for this purpose [15].

## 3. Results and Discussion

### 3.1. Simultaneous Determination of Linalool and Eugenol Content and FAs Profile in Basil Leaves

**Figure 1:**
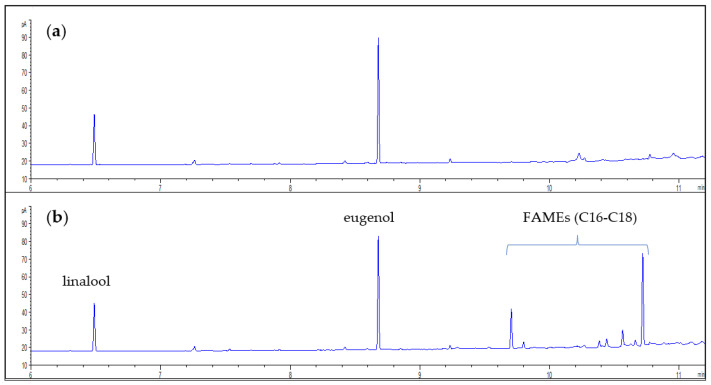
(**a**) Chromatogram obtained from the direct GC analysis of hexane extract; (**b**) Chromatogram obtained by the hexane extract after transmethylation.

The objective of the present study was to develop a method for analysing linalool and eugenol, usually representing two of the most abundant volatiles of basil leaves. The latter are known to contain not only volatile components, but also a fixed oil. This hampers the possibility of analysing directly by GC a solvent extract containing the volatiles from the leaves unless expecting a quick contamination of the chromatographic column and injector due to the presence of lipid components with consequent inefficiency of the instrumental system. However, acylglycerol derivatives could be transformed into FAMEs or into other volatile derivatives to be conventionally analysed by GC, providing information on the FA composition of the lipid fraction. In this study, we exploited this approach to combine the analysis of volatile components with the analysis of FAMEs in a single procedure. Basil leaf powder was extracted with hexane and the obtained extract containing both acyl lipids and volatile substances was subjected to transmethylation, avoiding any previous and subsequent evaporation of the solvent to prevent loss of volatile components. Figure 1 shows the chromatograms obtained analysing directly the hexane extract (Figure 1a) and the hexane extract after transmethylation (Figure 1b), respectively.

The last peaks appearing in the chromatograms (with retention times between 9.5 and 11.0 min) are referred to FAMEs, evidencing the presence of significant amounts of lipids in the extract and highlighting the importance of avoiding a direct injection in the GC of a solvent extract of the leaves. Chromatographic column used is a polar 50%-cyanopropylphenyl)-methylpolysiloxane column that allows a good resolution of FAMEs. However, in cases in which FAMEs are not of interest, apolar column (such as a 5% phenylpolydimethylsiloxane) can be successfully used for the analysis of volatile components, even using a faster oven temperature program, thus resulting in shorter GC chromatography run times. 

**Figure 2:**
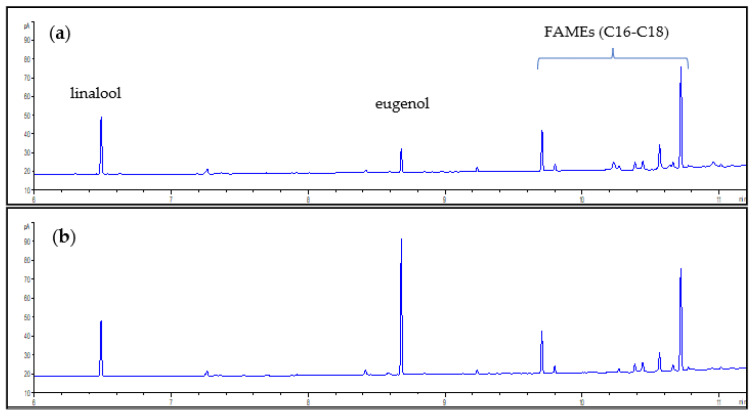
(**a**) Chromatogram obtained quenching the transmethylation reaction with water; (**b**) Chromatogram obtained by quenching the transmethylation reaction with acidic solution.

The proper quenching of the reaction is also important; an acidic aqueous solution is needed to avoid losses of phenolic compounds (or other acidic volatile components). Figure 2a reports the chromatogram obtained quenching the reaction with water, and Figure 2b the chromatogram obtained quenching the reaction with an acidic solution that neutralizes alkaline catalyst used for the transmethylation reaction. Eugenol recovery is significantly reduced (30%) when only water is used due to its loss in the form of phenolate salt in the aqueous phase.

### 3.2. Analytes Identification by GC-MS 

In order to ascertain the identity of substances detected, GC-MS analysis was also performed in one of the investigated samples using the same chromatographic column employed in the GC-FID analysis. The identified compounds are listed in Table 2.

A total of 16 compounds were detected and identified (Table 2). FAMEs abundance is significant, representing 56.6% of the total component detected in terms of % area and where linolenic acid was the most abundant (28.1%) followed by palmitic acid (14.9%). Taking into consideration the volatile compound composition, the most abundant substances were eugenol and linalool with percentage areas of 27.3 and 9.4%, respectively. Beyond linalool and eugenol, other volatile compounds were detected in low relative amount (0.3–1.7%). Most of them, such as eucalyptol, α-bergamotene, γ-cadinene, germacrene D, and α-terpineol, are aromatic compounds which are known to characterize the chemical composition of basil EOs [16,17]. The presence of phytol can be explained since it is the most abundant photosynthetic pigment present in green-leaved plants as a product coming from chlorophyll degradation [18].

### 3.3. Application of the Method to the Samples

The method was applied for the quantification of eugenol and linalool and for the simultaneous determination of the FA profile in 10 samples of powdered basil leaf samples by GC-FID analysis. Results for linalool and eugenol are reported in Table 3, while results for FAs are reported in Table 4.

Results show a higher prevalence of eugenol whose quantities are in the range of 2.50–2.99 g kg^−1^ of dry weight over linalool which is present as the second most abundant compound of the basil leaf volatile components and which was quantified in the range of 0.94–1.10 g kg^−1^, with a eugenol/linalool ratio in the range of 2.43–2.97. Most of the works reported in the literature are focused on the characterization of aromatic volatile compounds of basil leaf EOs. Linalool and eugenol are known to be among the most abundant components present in basil leaves, anyway; depending on the basil chemotype, the EOs components profile can vary greatly, as reported in the study by Shultz et al. [19], where the characterization of 11 different basil chemotypes was assessed showing that each one was characterized by a special chemical fingerprint made up of the most abundant substances of the plant volatile fraction. The ratio and abundance of aromatic components can be very variable depending on different factors such as cultivar, climate, year of cultivation, and others. Eugenol and linalool were also the two major components of the four basil varieties cultivated in Egypt in the study reported by Said-Al Ahl et al. [20]. They studied the EO components in order to classify the variety and the eugenol/linalool ratio varied in the range of 0.9–2.1, which is a little bit lower compared to our results. Mostly, when linalool and eugenol are the characterizing components of basil EOs, linalool is present at higher concentrations. As an example, Wogiatzi et al. [21] performed a study on selected basil cultivars and observed that broad leaf basil cultivar had a linalool/eugenol ratio of 7.6, while for narrow leaf basil it was much lower, 4. Hence, it can be concluded that the relative ratios of the two components is a very peculiar characteristic that can depend on a number of different parameters among which the variety plays an essential role. On the other hand, the sample treatments can also have a relevant impact on the aromatic components as reported by Díaz-Maroto et al. [22]. They performed the extraction of aromatic substances by simultaneous distillation/extraction (SDE) of basil samples and identified 27 components, identifying linalool and eugenol as the most abundant ones. They observed that the relative component quantities where different from fresh and dried samples and dependent also on the drying method, showing also how it reflects on sensory evaluations. In particular, for oven-dried samples (45 °C for 15 h) the mean concentrations of linalool and eugenol were 3.2 and 1.4 g kg^−1^, showing an opposite trend compared to our samples (1.01 ± 0.04 g kg^−1^ for linalool and 2.80 ± 0.15 g kg^−1^ for eugenol), confirming the high amount variability of volatile compounds.

The fatty acid profile of the samples under investigation was composed of 7 fatty acids from palmitic acid (C16:0) to arachidic acid (C20:0) whose composition, determined by the method developed in the present study, is detailed in Table 4 (hexane extraction). Linolenic acid (C18:3*n*3) was the most abundant with an average percentage area of 54.7%. The second FA in terms of relative abundance was palmitic acid (C16:0) with percentage areas up to 22.4%. In addition, linoleic acid showed rather high percentage areas in the range of 9.6–11.3% while the other fatty acids were present in much lower percentages. Essential fatty acids, namely linoleic and linolenic acids, represent a significant proportion of the FA profile, on average 65.1% of the total FA composition.

Linolenic acid, an *n*3 FA, is a very important PUFA, and its assimilation has been demonstrated to be essential for the maintenance of cardiac and cardiovascular health and for the prevention of atherosclerosis [23,24]. Linoleic acid (*n*6 FA) is also an important PUFA, but, along the fact that it seems to have beneficial effects due to the low-density lipoprotein (LDL) decreasing effect, its excessive intake has been demonstrated to be linked to pro-inflammatory properties [25,26]. The results are in overall accordance with the study reported by Tarchoune et al. [11] where the FA composition of basil leaves samples was determined. The study showed that generally PUFAs were present with the highest proportion compared to SFAs and MUFAs, with percentages of 78.7, 19.4 and 1.9%, respectively. The samples under investigation in the present study showed similar trend and percentages, displaying on average 65.1, 25.7 and 9.1%, respectively. Moreover, comparing the average percentages of the individual FAs, the results were similar for palmitic (16.2 and 20.6%), linolenic (69.0 and 54.7%) and linoleic acid (9.7 and 10.4%), where the second value in the bracket represents the average value found in the present study. On the contrary, in the study conducted by Vlaicu et al. [12] the most abundant fatty acids were SFAs followed by PUFAs and MUFAs (40.5, 36.6 and 21.9%, respectively), with palmitic and linoleic acids having comparable values (23.0 % and 17.4%, respectively) and linolenic acid having a much lower percentage (15.9%) as compared to the study reported by Tarchoune et al. [11] (69.0%) and to ours (54.7%). The different way samples have been prepared in the mentioned studies do not allow to establish the reason for the different FA composition found. Another important parameter is the PUFA *n*6/*n*3 (or ω6/ω3) ratio, since *n*6 and *n*3 are known to play important but opposite roles in inflammation modulation. A high value of this parameter is usually associated with degenerative pathologies [27]. In fact, while a high *n*6 dietary consumption seems to increase the concentrations of inflammatory mediators, high values of *n*3 are, by contrary, associated with the suppression effects on chronic diseases [28]. Hence, while ratios ranging from 20:1 to 15:1 have been seen to promote pathogenesis of many diseases, a ratio as close as possible to 1:1 is considered a healthier one [29]. From the FAs analysis of the samples under investigation the ratio obtained is in the range of 0.17–0.20, the result indicating the high-quality profile of the lipid fraction of basil leaves. Fatty acid profile obtained with hexane extraction has been compared with the fatty acid profile obtained by extracting the lipid fraction from the leaves by using the Folch method, where the combination of apolar and polar organic solvent, namely chloroform and methanol, allows to extract also polar lipids. Results obtained after derivatisation of acyl lipid components as FAMEs are reported in Table 4 (Folch extraction). The two methods provide extracts with comparable average percentages of palmitic acid, 20.6% for the hexane extract and 21.2% for the samples obtained from Folch method extraction and of palmitoleic acid (3.8 and 4.0%, respectively). For the other FAs, a significant difference (*p* < 0.001) by the two methods was obtained. Oleic and linoleic acids showed higher percentages in the hexane extract (5.6 and 10.4%, while for Folch 2.9 and 6.2%, respectively) and the ratio between the percentage areas obtained with the two methods is 1.89 for oleic and 1.66 for linolenic acid. Concerning linolenic acid, on the contrary, the higher percentage was obtained with the Folch method extraction, even if the difference was lower, with a Folch/hexane ratio of 1.14. These values could also be used as correction factors to convert the fatty acid composition obtained by the procedure reported in this paper into the composition resulting from Folch method, since these factors are rather reproducible in the different samples (relative standard deviations of the difference between the two methods vary from 0.0 to 7.6). These differences could be due to the amount of polar lipids extracted by the Folch method that could have a fatty acid composition quantitatively different than that of the neutral lipids, with are richer in PUFAs.

## 4. Conclusions

A time-saving method was validated for the simultaneous GC-FID analysis of bioactive compounds in basil leaves, namely eugenol, linalool, and fatty acids, affecting both sensorial and healthy properties of this plant-based food ingredient.

In the procedure proposed in the present paper, volatile components are extracted with hexane from the powdered basil leaf samples. The hexane extract is then subjected to derivatization to transform acyl lipids into FAMEs, thus allowing the subsequent direct GC-FID analysis of the major volatile compounds together with FAMEs. In none of the steps the solvent is evaporated, thus avoiding possible losses of volatile compounds. The major advantages of this method are: (i) the short time needed for sample preparation and analysis (a total of 40 min are needed); (ii) the avoidance of the isolation of the EO; (iii) the simultaneous determination of different classes of substances (the major volatile compounds and fatty acids); (iv) the use of very small amounts of organic solvents and (v) the use of analytical instrumentation commonly available in laboratories. 

The proposed method provides recovery rates of 105% for linalool and 97% for eugenol. Limits of quantification are 0.18 g kg^−1^ for linalool and 0.22 g kg^−1^ for eugenol, values that are much below the lowest concentrations found in the samples (2.50 and 0.94 g kg^−1^, for linalool and eugenol, respectively).

This method could be recommended for a fast routine quality control of basil leaves, used as food or also in food supplement formulations.

## Figures and Tables

**Table 1:** Linearity, LOD and LOQ referred to standard solution (µL L^−1^) and referred to the sample powder (g kg^−1^) and recovery of eugenol and linalool from basil samples.

	Linearity (µL L^−1^)	LOD (µL L^−1^)	LOQ (µL L^−1^)	LOD (g kg^−1^)	LOQ (g kg^−1^)	R^2^	Recovery (%)
linalool	20–400	9.2	30.6	0.05	0.18	0.9995	105
eugenol		11.2	37.3	0.07	0.22	0.9995	97

LOD: limit of detection; LOQ: limit of quantification; R^2^: linear regression coefficient.

**Table 2:** Compounds detected by the GC-MS analysis of a transmethylated basil leaves extract sample.

	Match Quality (%)	% Area	
Volatile components		43.4	% area (in total volatile components)
eucalyptol	99	1.2	2.6
linalool	86	9.4	21.4
α-terpineol	87	0.5	1.1
α-bergamotene	89	1.4	3.2
germacrene D	94	0.3	0.6
γ-cadinene	96	0.4	0.8
eugenol	98	27.3	63.1
(+)-*epi*-bicyclosesquiphellandrene	76	1.2	2.9
phytol	93	1.7	3.9
FAMEs		56.6	% area (in total FAMEs)
palmitic acid, methyl ester (C16:0)	98	14.9	26.3
palmitoleic acid, methyl ester (C16:1)	98	2.6	4.5
stearic acid, methyl ester (C18:0)	99	2.5	4.4
oleic acid, methyl ester(C18:1*n*9)	99	2.5	4.4
linoleic acid, methyl ester (C18:2*n*6)	99	5.4	9.5
linolenic acid, methyl ester (C18:3*n*3)	98	28.1	49.7
arachidic acid, methyl ester (C20:0)	97	0.6	1.1

Match quality (%) is referred to the comparison of experimental mass spectra with those found in NIST Library (2017).

**Table 3:** Eugenol and linalool content in the 10 samples analysed: concentration ranges, mean values, standard deviation (sd) and ratio between eugenol and linalool.

		Range	Mean ± sd
Eugenol	g kg^−1^ (dry weight)	2.50–2.99	2.80 ± 0.15
Linalool		0.94–1.10	1.01 ± 0.04
Ratio Eugenol/Linalool		2.43–2.97	2.73 ± 0.13

**Table 4:** Comparison between the % fatty acid composition obtained with hexane extraction and with Folch extraction: % range in the 10 samples analysed, mean and standard deviation (sd).

	Hexane Extraction			Folch Extraction		
	% Range	Mean	sd	% Range	Mean	sd
C16:0	19.3–22.4	20.6	0.8	19.1–23.9	21.2	1.8
C16:1	3.6–4.3	3.8	0.2	3.7–4.7	4.0	0.4
C18:0	3.4–4.5	3.7	0.3	1.7–2.7 ***	2.5	0.3
C18:1*n*9	4.7–7.3	5.6	0.8	2.2–3.3 ***	2.9	0.3
C18:2*n*6	9.6–11.3	10.4	0.4	5.7–6.6 ***	6.2	0.2
C18:3*n*3	52.1–56.1	54.7	1.7	59.8–64.9 ***	62.7	2.0
C20:0	0.8–1.8	1.2	0.3	0.3–0.6 ***	0.4	0.1
SFA	24.0–27.6	25.7	1.1	22.2–26.7 *	24.2	1.6
MUFA	8.5–9.6	9.1	0.7	5.9–7.9 ***	6.9	0.6
PUFA	62.9–67.5	65.1	1.6	65.8–71.1 ***	68.9	2.0
Ratio *n*6/*n*3 PUFA	0.17–0.20	0.19	0.0	0.09–0.11 ***	0.10	0.0

SFA, Saturated Fatty Acids; MUFA, Mono-Unsaturated Fatty Acids; PUFA, Poly-Unsaturated Fatty Acids. Significant differences (One-way ANOVA) between the two extraction methods are indicated for each FA by * (*p* < 0.05), *** (*p* < 0.001).

## Data Availability

The data used to support the findings of this study can be made available by the corresponding author upon request.
